# *ptk2* and *mt2a* Genes Expression in Gastritis and Gastric Cancer Patients with *Helicobacter pylori* Infection

**DOI:** 10.1155/2022/8699408

**Published:** 2022-08-25

**Authors:** Manouchehr Ahmadi Hedayati, Delniya Khani, Farshad Sheikhesmaeili, Bijan Nouri

**Affiliations:** ^1^Liver and Digestive Research Center, Research Institute for Health Development, Kurdistan University of Medical Sciences, Sanandaj, Iran; ^2^Department of Microbiology, Faculty of Medicine, Kurdistan University of Medical Sciences, Sanandaj, Iran; ^3^Department of Epidemiology and Biostatistics, Faculty of Medicine, Kurdistan University of Medical Sciences, Sanandaj, Iran

## Abstract

**Background:**

*ptk2* and *mt2a* genes contribute to the cell cycle during proliferation and apoptosis, respectively. Designing a case-control study including gastric adenocarcinoma and gastritis patients with and without *Helicobacter pylori* infection would lead to determinate of the correlations between *ptk2* and *mt2a* genes expression with *H. pylori* infection in gastric antral epithelial cells.

**Methods:**

Overall, 50 and 30 gastric antral biopsy samples of gastric cancer (case group) and gastritis (control group) patients were included into study, respectively. All biopsy samples were collected considering the exclusion criteria including patients with a history of consumption of tobacco, alcohol, and anti-*H. pylori* drugs. Each patient group is divided into with and without *H. pylori* infection to detect cDNA fold changes of *ptk2* and *mt2a* genes by using Real Time RT PCR. Furthermore, the presence of *H. pylori* virulence genes was detected directly by using specific primers and simple PCR on cDNA synthesized from total RNA of gastric antral biopsy samples.

**Results:**

A negative correlation was revealed between age and clinical manifestations with the ΔCt value of the *ptk2* gene (*P* < 0.05). The *H. pylori iceA1/2* and *cagE* genes revealed positive and negative correlations with the ΔCt value of the *ptk2* gene (*P* < 0.05), respectively. Furthermore, a weak correlation was detectable between *H. pylori babA2/B, oipA*, and *cagY* genes and the ΔCt value of the *mt2a* gene in gastric antral epithelial cells of patients (*P* < 0.1).

**Conclusions:**

The results of the current study opened a view for more investigation on the stunning roles of *H. pylori* infection in clinical outcomes through *mt2a* and *ptk2* gene expression in gastric antral epithelial cells.

## 1. Introduction

In a close competition between carcinogenic agents and natural anti-cancer drugs such as *Allium*, cell signaling molecules are considered the effective targets [1]. In this regard, it has been revealed that the main pathways of natural anticancer drugs are to induce apoptosis by activating the MAPK and PI3K/AKT signaling pathways suppressing tumor growth [1]. In this meantime, Mt2a (Metallothionein 2A) as a member of the metallothionein proteins family contributes to critical roles including increasing intracellular concentration of heavy metals, protecting the cells against the toxicity of hydroxyl free radicals and heavy metals affecting cell apoptosis, proliferation, angiogenesis, and anti-inflammatory processes [2–4]. As a critical role in cell cycling, Metallothioneins control HMBOX1 (Homeobox containing 1) protein bound to the double-stranded repeat sequence of telomeres following regulation of zinc intracellular concentration [5–8]. Recent studies have shown that following LPS-induced inflammation and ROS production, HMBOX1 inhibits NF-*κ*B and the MAPK signaling pathway [1, 9]. In this regard, MT2A protein exerts its anti-cancer effects through inactivation of NFKBIA (NFKB Inhibitor Alpha) subsequent binding to MZF1 (Myeloid Zinc Finger 1) [9, 10]. As a clinical impact of Mt2a, it has recently been shown that attenuation of MT2A and IkB-*α* gene expression in human gastric epithelial cells is associated with a poor prognosis in gastric cancer patients [9, 10]. Furthermore, some *mt2a* gene polymorphisms, including rs28366003 and rs1610216, are associated with an increased risk of cancer [11–13]. In this regard, some MT-2A gene polymorphisms are associated with adenocarcinoma risk in the Iranian patient population [14]. Moreover, in the Polish population, the rs28366003 SNP polymorphism has been associated with breast and prostate cancer [15].

On the other hand, the *ptk2* gene encodes a cytoplasmic tyrosine kinase protein, namely, FAK (Focal Adhesion Kinase) [16–18]. The FAK/Paxillin signaling pathway conducts a substantial effect on the cell migration following activating SH2 domains of the Src kinase family and consequentially triggering downstream-activating signals leading to the regulation of cell motility, invasion, survival, and proliferation [19]. The changes in *ptk2* gene expression are associated with the pathological stage, progression, and cancer's specific survival [20]. There is evidence to support the role of FAK in promoting malignant phenotypes (unregulated cell proliferation, survival, and migration) of various tumor cells in vitro [21, 22]. The *ptk2* gene over-expression is commonly seen in metastatic cancers and is obviously associated with malignant clinical outcomes [23–25]. Furthermore, inhibitors of FAK kinase exert suppressing cancer cell growth in clinical trials [26–28]. In the other studies, the *ptk2* gene over-expression is well-known as a pathological risk factor predicting aggressive biological behavior of bladder carcinoma cells [29]. Considering a previous study, FAK is highly expressed in human gastric cancer cells and correlated with tumor progression, invasion, and metastasis [30]. To conclude, the studies show that the Fak protein and its gene [*ptk2*] are significantly associated with cancer progression [31, 32].

*H. pylori* as the most common causative agent of chronic infections in humans shows a full long history related to clinical implications from gastritis to gastric cancer [33]. In this regard, the studies show that the *H. pylori* virulence factors have an indispensable impact on the clinical consequences of *H. pylori* persistent active infection [33–37]. In the meantime, the epidemiological studies show that *H. pylori* adhesins, including Sab, Bab, and the type 4 secretory system (T4SS), are related to peptic ulcers and gastric cancer [33–36]. CagA suppresses the apoptotic activity of VacA and activates the signaling pathway of the factor NF-KB inducing overexpression of anti-apoptotic agents [33]. On the other hand, CagY and CagL proteins, as the significant members of T4SS proteins with a tubular structure, following contact with gastric epithelial cell surface receptors, including beta-1 integrin receptors, inject CagA carcinogen protein into the gastric epithelial cell [37–40]. Therefore, it is worth to mention that despite the presence of the *cagA* gene in the *H. pylori* strains genome, due to possible changes in the *cagY* gene expression under the full stress environment of the stomach, the CagA protein could not be able to inject into host cells [39]. This study investigated the correlation between the *H. pylori* virulence genes and *ptk2* and *mt2a* genes expression in gastric antral epithelial cells of gastritis and gastric cancer patients with *H. pylori* infection.

## 2. Materials and Methods

### 2.1. Sampling

A case-control study was designed on patients who had involved gastric adenocarcinoma and gastritis [41, 42]. Considering the prevalence of gastric adenocarcinoma and *H. pylori* infection in Sanandaj city, located in the west of Iran, using Cochran's formula, 30 and 50 patients with gastric adenocarcinoma and gastritis were registered [42]. Patients with a history of anti-*H. pylori* chemotherapy, alcohol, and cigarette consumption were excluded from the study. According to the study's aim, to investigate *H. pylori* infection's effect on the *mt2a* and *ptk2* genes expression, the gastric biopsy samples were collected from patients referred to Tohid Hospital in Sanandaj city. The *H. pylori* active infection was detected by using a urea breath test [43, 44]. Two gastric biopsy specimens were obtained from each patient by a gastroenterologist. One biopsy sample was prepared from the gastric antrum, the natural site of *H. pylori* infection, to detect *H. pylori* virulence genes and study *mt2a* and *ptk2* gene expression using PCR, and another biopsy from the gastric adenocarcinoma tumor area to pathologic evaluation. The gastric antral biopsy sample for molecular analysis was immediately dropped into RNA Later solution and transferred to the Molecular Microbiology Laboratory of Kurdistan University of Medical Sciences. On the other hand, the gastric tumor area biopsy samples including cardia, body, or pyloric area, were evaluated at the pathology center of Tohid Hospital.

### 2.2. PCR

First of all, total RNA was extracted from gastric antral biopsy samples (Miniprep Kit; Bio Basic Company, Toronto, Canada) and immediately converted to cDNA (Prime Script TM RT-PCR Kit; Takara Company, Japan). To survey the presence of the *H. pylori* genome in gastric antral biopsy samples, *H. pylori 16s rRNA* gene-specific primer and PCR method were used. Primers of *H. pylori* virulence genes were designed by using primer3 online software and blast on the PubMed website and then were synthesized by Bioneer company (South Korea; Tables 1 and 2). The thermocycler program was set based on the current protocols. Using Takara kits (Emerald Amp® MAX PCR Master Mix), PCR master mix was prepared. Single PCR steps were performed according to the current PCR protocols and using annealing temperatures of primers in a final volume of 25 microliter (Eppendorf® Co.; Tables 1 and 2) (17). Based on agarose gel electrophoresis protocols, PCR products were run on 1% agarose gel to observe PCR results. To prove the *H. pylori* genome, the *H. pylori vacA m2* gene was sequenced and registered in the GenBank with accession number MK642592.1.

### 2.3. Real-Time RT PCR

To detect *mt2a* and *ptk2* genes' expression in the gastric biopsy samples by using the Real-Time RT PCR method (Corbett machine, Co.), the specific primers of *mt2a* and *ptk2* as the target genes and GAPDH as the internal genes were designed by using primer3 software and their genes sequences in the GenBank (Table 3). Takara kits (Emerald Amp® MAX PCR Master Mix) were used to prepare the PCR master mix. The PCR amplification program included a 10 minutes denaturation step at 95°C and 40 cycles of denaturation at 95°C for 15 seconds, specific annealing temperatures for 30 seconds, and extension at 72°C for 60 seconds. The relative quantity of the *mt2a* and *ptk2* genes expression in gastritis (control group) and gastric adenocarcinoma (case group) patients were calculated by ΔCt (threshold cycle) = Ct (DNMT1) Ct (GAPDH). The mean of Ct of each 3 PCR reactions was put on the ΔΔCt formula to compute the fold change of each gene in the case and control groups by using the 2^−ΔCt^ formula [43–45].

### 2.4. Statistical Analyses

Quantitative and qualitative data analysis was performed using SPSS software version 26. Crosstab chi-square tests were used to survey the correlation and relationship between qualitative data [41, 42]. First, the quantitative data distribution status was determined using Kolmogorov–Smirnov statistical test [42]. The distribution of *mt2a* and *ptk2* genes ΔCt was abnormal. Accordingly, Mann–Whitney and Kruskal–Wallis tests were used to investigate the correlation between quantitative data and comparison in the case and control groups and subgroups [42]. The significance level was considered less than 0.05 [41].

## 3. Results

Based on the urea breath test results, 46% and 36.66% of gastritis and gastric adenocarcinoma patients showed *H. pylori* active infection, respectively (Table 4). It revealed that because of an anti-*H. pylori* drug without medical registration, the prevalence of *H. pylori* active infection among gastric adenocarcinoma patients was significantly lower than in gastritis patients (Table 4). Like in the previous studies, the frequency of *H. pylori* infection and gastric adenocarcinoma increased along with increasing age (*P* < 0.001; Tables 4 and 5).

Besides the genetic factors, exposure to environmental carcinogens factors, diet and *H. pylori* infection contribute to increasing gastric cancer risk [43, 44]. In this regard, although the results of the current study showed that the prevalence of gastric adenocarcinoma is significantly higher in men (*P* < 0.05; Table 4), it is worthy of note that this significant difference was not related to *H. pylori* infection (Table 5).

**Figure 1:**
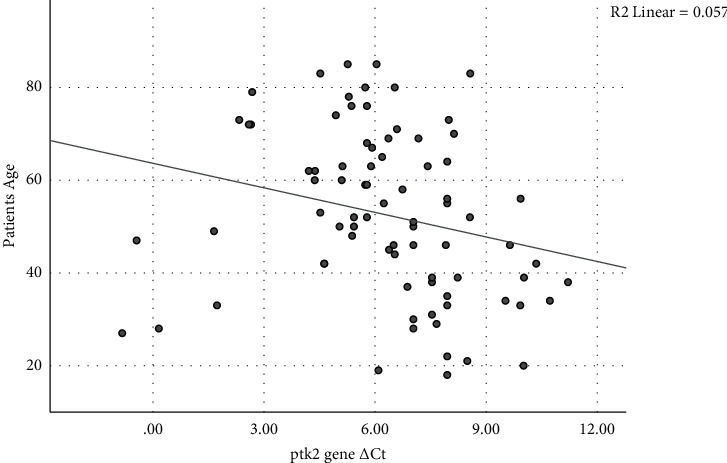
A simple scatter plot of ΔCt of the *ptk2* gene depicts a reverse relationship with *R*2 linear 0.057. This means the *ptk2* gene expression will increase along with increasing age.

The changes of *ptk2* and *mt2a* genes ΔCt in demographic subgroups indicated a negative correlation between *ptk2* gene ΔCt with patients' age and clinical manifestations (Table 6 and Figure 1). This means that the *ptk2* gene expression in gastric antral epithelial cells increases with getting older and gastric cancer up 2.51 and 4.32 times, respectively (*P* < 0.005). In addition, a significant decrease of ΔCt of the *ptk2* gene was revealed in patients with *H. pylori cagE*^+^ strains infection (Table 7). In these cases, *ptk2* gene expression increased 5.06-fold compared to *cagE*^−^ samples (*P* < 0.05; Table 8). On the other hand, gastric antral biopsies with infection of *H. pylori iceA1/2*^+^ strains showed a positive correlation with the ΔCt of the *ptk2* gene (*P* < 0.005). In samples with *iceA1/2*^*+*^ cDNA, the expression level of the *ptk2* gene increased by 7.66-fold than in samples with *iceA2*^+^ cDNA (*P* < 0.005).

Although no correlation was observed between *H. pylori* infection and ΔCt of *ptk2* and *mt2a* genes, a weak correlation was observed between *mt2a* gene ΔCt and subgroups with *babA2/B*, *oipA*, and *cagY* cDNA (*P* < 0.1; Table 9). In samples with *babA2/B*^*+*^ cDNA, the expression of the *mt2a* gene increased 5.14-fold than in samples with *babA2/B*^*-*^ cDNA (*P* < 0.005; Table 9).

## 4. Discussion

MT2A, as an oxidative shock protein protecting cell DNA, influences the cell cycle through apoptosis [2–4]. Pan et al. showed that overexpression of the *mt2a* gene is associated with increasing tumor grade and poor gastric cancer prognosis [10]. They showed that MT2A induces apoptosis by stopping the G2/M phase of the cell cycle, and the effect of MT2A on apoptosis is due to the suppression of the NF-KB signaling pathway [9, 10]. Wang et al. showed that MT2A increases the expression of P21 and BAX proteins by affecting P53 which is a pre-apoptotic protein triggering the cell death process [12].

Previous studies show the positive correlations between *mt2a* gene polymorphisms and breast, liver, and prostate cancers [11–15]. In a comparison, the results of the current study showed no statistically significant correlation between *mt2a* gene ΔCt and patients' demographic characteristics, including clinical manifestations, tumor grade, adenocarcinoma tumor area, age, sex, and *H. pylori* infection (Table 5). On the other hand, weak and negative correlations were observed between the cDNA of *babA2/B*, *oipA*, and *cagY* virulence genes of *H. pylori* with *mt2a* gene ΔCt (*P* < 0.1; Table 7). The *mt2a* gene expression in gastric biopsy samples with *H. pylori babA2/B*^*+*^ cDNA was higher up to 5.14-fold than in biopsy samples with *H. pylori babA2/B*^*-*^ cDNA (Table 9). These results indicate that the simultaneous expression of *H. pylori babA2*^*+*^ and *babB*^*+*^ genes (*babA2/B*^*+*^ cDNA) correlates weakly with increased *mt2a* gene expression (*P* < 0.1). Furthermore, previous studies have shown the negative and positive correlations between gastric cancer with *H. pylori babA2/B* and *cagE*, *cagA*, and *iceA1* genes expression, respectively (Table 9).

Aras et al. showed that the *cagY* gene has two reproducible and variable regions at the 5′ end sequence including forward (FRR) and middle (MRR) [38]. They showed that following an increase or decrease in the copy number of reproducible sequences, the *cagY* gene expression and consequently CagY protein's efficiency to bind to *β*1 integrin receptors of gastric epithelial cells will change [38]. They revealed that changes in the CagY protein antigens would give a trait to bacteria to escape from the gastric mucosal immune system and establish a persistent infection [38]. The present study showed a statistically weak correlation between the *H. pylori cagY* gene with the MRR region and increased mt2a gene expression up to 2.2 times (*P* < 0.1; Table 9). The *mt2a* gene expression in the biopsy specimens with the *H. pylori cagY-MRR*^+^ cDNA was higher than in the biopsy specimens with the *H. pylori* cagY-MRR^−^ cDNA (*P* < 0.1).

FAK protein is known as a tyrosine kinase integrin that its gene expression will increase along with cancer progression [19–22]. Some studies have shown a gradual increase in FAK protein (*ptk2* gene) related to poor cancer prognosis [29]. Zhang et al. showed a high *ptk2* gene expression correlated with aggressive traits of bladder squamous cell carcinoma [29]. Their Kaplan–Meyer analysis also showed a correlation between *ptk2* gene expression and the pathological stages, invasion of the disease, and reduced chance of survival in carcinoma [29]. The present study showed that the *ptk2* gene expression and the frequency of gastric cancer increase along with increasing age (*P* < 0.001).

Previous studies, based on the bacterial effects on the host cytoskeleton, show Hijacking of the FAK/Paxillin pathway to control cell physiological trends [51]. In this regard, Kim et al. showed following being attached *Shigella flexneri* OspE protein to integrin-binding kinase, the integrin B1 gene expression will be increasing, and suppression occurs as a result of phosphorylation of FAK and Paxillin [51].

## 5. Conclusion

In a remarkable conclusion, this study showed that the gastric antral epithelial samples with infection of *H. pylori iceA1/2* and *cagE* genotypes reveal a strong correlation with increased *ptk2* gene expression (*P* < 0.001).

## Figures and Tables

**Table 1:** Primers of *H. pylori* T4SS, *16s rDNA,* and *vacA* in simple PCR.

Specific primers	Sequence	Annealing Tm°C	Product size bp	Reference
*16s rDNA H. pylori* F *16s rDNA H. pylori* R	CTGGAGAGACTAAGCCCTCC AGGATCAAGGTTTAAGGATT	50	446	This study

*cagA* F *cagA* R	TGACCAACAACCACAAACCG TCAGGATCGTATGAAGCGACAG	57	108	This study

*cagA* EPIYA-C F *cagA* EPIYA-C R	AAGAAAGCAGGACAAGCAGC CTAACCGATCGCCCTACCTT	55	188	This study

*cagT* F *cagT* R	AGGGTGTGGTGATGATAGCG TGCTTGTTGTTTGCTCCACT	55	154	This study

*cagE* F *cagE* R	GAATGGAGCGAGCGATGAAA TAGGAATTTGCAGCGCTCAC	56	163	This study

*cagY* F *cagY* R	AGTTCAAGTGGCGCTAGATTG ACAAGCCTTTCAAGCATTCGT	57	200	This study

*vacA* s1/s2 F *vacA* s1/s2 R	ATGGAAATACAACAAACACAC CTGCTTGAATGCGCCAAAC	55	259/286^*∗*^	Atherton et al. [46]

*vacA* m1/m2 F *vacA* m1/m2 R	TGGATAGTGCGACTGGGTTT TCCATGCGGTTGTTGTTGTT	54	205/220^*∗*^	This study

^*∗*^The primers yield various fragments of *vacA* depending on the presence of a repetitive nucleotides sequence in some *vacA* alleles.

**Table 2:** Primers of *H. pylori* adhesins and surface proteins.

Specific primers	Sequence	Annealing Tm°C	Product size bp	Reference
*iceA1* F *iceA1* R	GTGTTTTTAACCAAAGTATC CTATAGCCASTYTCTTTGCA	45	247	Van Doorn et al. [47]

*iceA2* F *iceA2* R	GTTGGGTATATCACAATTTAT TTRCCCTATTTTCTAGTAGGT	47	229/234	Van Doorn et al. [47]

*hopQI* F *hopQI* R	ACGAACGCGCAAAAACTTTA TTGCCATTCTCATCGGTGTA	55	187	Sicinschi et al. [48]

*hopQII* F *hopQII* R	ACAGCCACTCCAATCCAGAA AACCCCACCGTGGATTTTAG	55	160	Sicinschi et al. [48]

*babA2* F *babA2* R	CAATGCGGTGCGTGAAAATC ATACCCTGGCTCGTTGTTGA	57	205	This study

*babB* F *babB* R	CAATTCCCCGGCGTATCAAG ATTGCAAGTGATGGTCGTCG	56	175	This study

*sabA* F *sabA* R	TCTCTCGCTTGCGGTATCAT AGCTCAATGTTGTTGGCGTT	56	204	This study

*sabB* F *sabB* R	GCATTCAAACGGCGAACAAC TCCTGTGCAGTTCCCATCTT	56	248	This study

*alpA* F *alpA* R	CGCTCCTATCAAAACCGCTC TTCCCGTCCAACTTACCGAA	55	185	This study

*alpB* F *alpB* R	TCAACTTGCGAGCAGACCTA AGCCATAGACCCCATACACG	57	218	This study

*oipA* F *oipA* R	CTCCACGCTGAAAGGAATGG CCATTTCCTGCGAATCGGTT	55	233	This study

**Table 3:** Specific Primers of *ptk2* and *mt2a* in Real-Time RT PCR.

Specific primers	Sequence	Annealing Tm°C	Product size bp	Reference
*GAPDH* F *GAPDH* R	GAAGGTGAAGGTCGGAGTCAAC CAGAGTTAAAAGCAGCCCTGGT	60	71	
*ptk2* F *ptk2* R	GCACCCACCGAGAGATTGAG GGGCCAGTTTCATCTTGTTGA	60	81	[49]
*mt2a* F *mt2a* R	GCCCAGGGCTGCATCTG TTTGTGGAAGTCGCGTTCTTTA	60	102	[50]

**Table 4:** Demographic data of patients.

	Gastritis	Gastric cancer	Total	*P* value
*H. pylori* infection (positive/negative)	23/27	11/19	34/46	0.487
Sex (male/female)	24/26	24/6	48/32	0.024
Age group (18–30/31–45/46–60/61–85)	10/18/17/5	0/0/7/23	10/18/24/28	0.005

**Table 5:** The frequency of *H. pylori* infection among males and females in various age groups.

*H. pylori* infection	Sex (*n*: 80)		Age group (*n*: 80)			
	Male	Female	18–30	31–45	46–60	61–85
*H. pylori* infection (positive, *n*: 34)	19	15	2	10	11	11
*H. pylori* infection (negative, *n*: 47)	29	17	8	8	13	17
Total	48	32	10	18	24	28

**Table 6:** Correlations between *ptk2* and *mt2a* ΔCt and patients' demographic characters.

	Sex	Age group	Disease	*H. pylori* infection	Gastric cancer area	Tumor grade
*ptk2* correlation *P* value	0.080 0.589	−0.334 0.002	−0.379 0.001	0.042 0.779	−0.107 0.636	0.345 0.116
*mt2a* correlation *P* value	0.146 0.323	−0.055 0.710	−0.104 0.482	−0.049 0.742	0.041 0.858	0.257 0.248

**Table 7:** Correlations between *ptk2* and *mt2a* ΔCt and gene expression of *H. pylori* virulence genes in gastric antral epithelial cells of gastritis and gastric adenocarcinoma patients.

	*vacA s1m1/s1m2*	*cagA*	*cagA- EPEAYC*	*cagT*	*cagY*	*cagE*	*sabA/B*	*babA2/B*	*hopQI/II*	*alpA/B*	*oipA*	*iceA1/2*
*ptk2* correlation *P* value	0.320 0.065	−0.216 0.312	−0.156 0.468	−0.082 0.804	−0.238 0.262	−0.390 0.023	−0.215 0.314	0.192 0.370	−0.192 0.368	0.186 0.384	0.152 0.472	0.519 0.002
*mt2a* correlation *P* value	−0.256 0.227	0.292 0.166	0.304 0.149	0.009 0.966	−0.378 0.068	0.289 0.171	−0.367 0.087	−0.388 0.061	0.040 0.854	−0.093 0.665	−0.348 0.095	−0.293 0.165

**Table 8:** The statistical relationships of *ptk2* genes expression with *H. pylori* infection and its virulence genes expression using Mann–Whitney *U* test. Fold changes of ptk2 gene expression were calculated by using the average of *ptk2* ΔCt in the case and control groups (*P* < 0.05).

		*N*	Mean rank	*Z*	*P* value	Mean ΔCt *ptk2*	Fold change
Disease	Adenocarcinoma Gastritis	30 50	29.20 47.28	−3.371	0.001	5.4730 6.8048	2.51
Age group	61–85 31–45	28 18	18.48 31.31	−3.163	0.002	5.6203 7.7354	4.32
*iceA1/2*	*iceA2iceA1/2*	17 17	12.4122.59	−2.983	0.002	4.8719 7.8093	7.66
*cagE*	Positive Negative	5 29	8.3 19.09	−2.239	0.023	4.3460 6.6845	5.06

**Table 9:** The statistical results of *mt2a* gene expression with *H. pylori* infection and its virulence genes expression using Mann–Whitney *U* test. Fold changes of *mt2a* gene expression were calculated by using the average of *mt2a* ΔCt in the case and control groups (*P* < 0.01).

		*N*	Mean rank	*Z*	*P* value	Mean ΔCt *mt2a*	Fold change
*babA2/B*	Negative *babA2/B*	17 3	9.08 3.67	−1.876	0.061	5.3767 3.0167	5.14

*oipA*	Negative Positive	3 31	20.50 11.77	−1.671	0.095	6.1300 4.6918	2.71

*cagY*	*CagY-MRR* negative	13 21	9.40 14.71	−1.815	0.068	4.1490 5.2850	2.2

## Data Availability

All data are already released in the article results and the raw datasets used and/or analyzed during the current study are not publicly available due to contracts with research participants but are available from the corresponding author upon reasonable request.
